# Amyloid accelerates tau propagation and toxicity in a model of early Alzheimer’s disease

**DOI:** 10.1186/s40478-015-0199-x

**Published:** 2015-03-24

**Authors:** Amy M Pooler, Manuela Polydoro, Eduardo A Maury, Samantha B Nicholls, Snigdha M Reddy, Susanne Wegmann, Christopher William, Lubna Saqran, Ozge Cagsal-Getkin, Rose Pitstick, David R Beier, George A Carlson, Tara L Spires-Jones, Bradley T Hyman

**Affiliations:** Department of Neurology, Massachusetts General Hospital, Harvard Medical School, 114 16th Street, Charlestown, MA 02129 USA; Department of Basic and Clinical Neuroscience, Institute of Psychiatry, Psychology & Neuroscience, King’s College London, DeCrespigny Park London, SE5 8AF UK; McLaughlin Research Institute, Great Falls, MT 59405 USA; Center for Developmental Biology and Regenerative Medicine, Seattle Children’s Research Institute, Seattle, WA USA; University of Edinburgh, Centre for Cognitive and Neural Systems, 1 George Square, Edinburgh, EH8 9JZ UK

**Keywords:** Neurofibrillary tangles, Amyloid plaques, Alzheimer’s disease, Tauopathy, Aggregation

## Abstract

**Introduction:**

In early stages of Alzheimer’s disease (AD), neurofibrillary tangles (NFT) are largely restricted to the entorhinal cortex and medial temporal lobe. At later stages, when clinical symptoms generally occur, NFT involve widespread limbic and association cortices. At this point in the disease, amyloid plaques are also abundantly distributed in the cortex. This observation from human neuropathological studies led us to pose two alternative hypotheses: that amyloid in the cortex is permissive for the spread of tangles from the medial temporal lobe, or that these are co-occurring but not causally related events simply reflecting progression of AD pathology.

**Results:**

We now directly test the hypothesis that cortical amyloid acts as an accelerant for spreading of tangles beyond the medial temporal lobe. We crossed rTgTauEC transgenic mice that demonstrate spread of tau from entorhinal cortex to other brain structures at advanced age with APP/PS1 mice, and examined mice with either NFTs, amyloid pathology, or both. We show that concurrent amyloid deposition in the cortex 1) leads to a dramatic increase in the speed of tau propagation and an extraordinary increase in the spread of tau to distal brain regions, and 2) significantly increases tau-induced neuronal loss.

**Conclusions:**

These data strongly support the hypothesis that cortical amyloid accelerates the spread of tangles throughout the cortex and amplifies tangle-associated neural system failure in AD.

**Electronic supplementary material:**

The online version of this article (doi:10.1186/s40478-015-0199-x) contains supplementary material, which is available to authorized users.

## Introduction

Progressive accumulation of protein aggregates throughout the brain is a hallmark of Alzheimer’s disease (AD). Amyloid plaques, distributed throughout neocortical areas [27], occur in perhaps a third to half of individuals over age 65 [15,3]. Tau inclusions, called neurofibrillary tangles (NFT), occur nearly universally in individuals over the age of 65, but are nearly always limited to entorhinal cortex (EC) and closely associated regions unless amyloid plaques also occur, when the pattern of tangles can extend to other limbic areas and association cortices [3,11,1]. The extent of symptoms due to Alzheimer’s disease is closely correlated with the anatomical extent and number of tangles in the cortex [12]. This set of observations led us to pose two alternative hypotheses: 1) the presence of plaques is permissive for, or at least a critical enhancer of, the mechanism that leads to spread of NFT to the cortex, or 2) NFT and plaque evolution occurs as the disease progresses, but their progression is not causally related to one another.

Recent data from the rTauEC transgenic mouse model in which human P301L mutant tau overexpression uniquely in the EC leads, at advanced age (24 months), to the appearance of numerous human tau containing NFT in synaptically connected brain regions suggests that tau spreading can occur by transfer of misfolded human tau protein between neurons [6,10,16]. This model affords an opportunity to directly test the hypothesis that cortical amyloid deposits accelerate the spread of NFT across cortical areas. We crossed rTgTauEC mice with a model that exhibits wide-spread cortical Aβ plaque deposition to produce a APP/PS1 x rTauEC line. We examined brains at 16 months, an age prior to the expected tau propagation phenotype, and found substantial human tau propagation from the EC to synaptically-connected regions in the rTgTauEC x APP/PS1 mice, compared to almost no propagation of NFT (at this age) in the parental rTgTauEC line. In addition, the rTgTauEC x APP/PS1 mice exhibited exacerbated neuritic dystrophies, and importantly, degeneration of tau expressing neurons in the EC, a substantially more severe phenotype than observed in age-matched rTauEC mice. The data presented here support the hypothesis that the presence of amyloid in the neocortex acts as an accelerant for tau toxicity and propagation.

## Materials and methods

### Experimental design

The objective of this study was to determine cortical amyloid deposition acts as an accelerant for spreading of neurofibrillary tangles beyond the medial temporal lobe in AD. We generated a new transgenic mouse model of AD by crossing mice expressing pathological human tau in the entorhinal cortex with APP/PS1 mice which develop widespread amyloid plaques. At 10- and 16-months of age, we quantified human tau-positive cell bodies, amyloid plaques, cholinergic fiber sprouting, cell death and axonal dystrophy in brains of these mice. We also quantified these parameters in mouse lines that expressed only localized human tau, APP/PS1, or control littermates expressing only the activator transgene. For all *in vivo* experiments, *n* values reported are individual animals.

### Animals

Several transgenic mouse lines were used in this study. Mice with regulatable P301L human tau in the entorhinal cortex (rTgTauEC) have been characterized previously [6]. APPswe/PS1dE9 mice (APP/PS1) were obtained from Jackson Laboratory (stock line B6.Cg-Tg(APPswe,PSEN1dE9)85Dbo/J). The generation of mice expressing both APP/PS1 and rTgTauEC (rTgTauEC x APP/PS1) was described previously [23]. Incipient B6 congenic (4^th^ or 5^th^ backcross generation with DBA/2 J passenger loci) “S-line” mice were mated to B6.APP/PS1, which carries C3H/HeJ passengers. Mice positive for both the EC-tTA and APP/PS1 transgenes arrays were then crossed to FVB-Tg(Tau_P301L_)4510 mice to produce rTgTauEC x APP/PS1 mice. Brains from gender-mixed 10- and 16-month-old rTgTauEC, APP/PS1 and rTgTauEC x APP/PS1 mice were used in the present study. As stated above, age-matched littermates expressing only the activator transgene were used as human tau-negative controls. All animal experiments conformed to United States National Institutes of Health guidelines and were approved by the Institutional Animal Care and Use Committees of Massachusetts General Hospital and McLaughlin Research Institute. This article does not contain any studies with human participants performed by any of the authors.

### Immunohistochemistry

Mice were sacrificed by CO_2_ inhalation and brains were frozen and embedded in M1 mounting medium (Shandon, Thermo Scientific). 10 mm thick horizontal brain tissue sections were cut on a cryostat, mounted on glass slides and stored at −80 C. For immunofluorescence labeling, sections were fixed in PBS containing 4% paraformaldehyde for 10 min before being permeabilized in 0.1% Triton solution (20 min) and blocked in 5% normal goat serum (NGS) for 1 h. The following primary antibodies were diluted in PBS containing 1% NGS: Tau13, monoclonal anti-human tau (1:500), Covance; Alz50, monoclonal anti-tau, conformation-dependent (1:100), courtesy of Peter Davies; AW7, polyclonal anti-Aβ (1:5000), courtesy of Dominic Walsh; glial fibrillary acidic protein (GFAP; 1:1000), Sigma; and SMI312, monoclonal anti-neurofilament (1:5000). Sections were incubated in the appropriate antibody mixture overnight at 4°C, and then washed thoroughly in Tris-buffered saline (TBS) before incubation in the appropriate secondary antibody (1:500), in 1% NGS for 1 h at room temperature. Secondary antibodies were fluorescent anti-mouse or anti-rabbit Alexa Fluor 488 (Life Technologies), Cy3-labeled or Cy5-labeled (Jackson ImmunoResearch Laboratories). Sections were counterstained with DAPI and mounted using antifade mounting medium (VectaShield). Images were recorded on a Zeiss AxioImager epifluorescence microscope equipped with a Coolsnap digital camera and Axio-Vision v4.8 software.

### Cell quantification

To determine whether accumulation of tau and Aβ-induced cell loss in the EC, cell nuclei were quantified in brains of rTgTauEC and rTgTauEC x APP/PS1 mice at 16 months of age. In four sections for each animal, DAPI labeled nuclei in layer II of the EC were counted applying thresholding and particle counting plugins using Fiji (National Institutes of Health).

### Acetylcholinesterase assay

To visualize cholinergic fibers in the DG, 10 mm frozen brain sections mounted on slides were brought to room temperature and incubated overnight in 0.68% sodium acetate buffer (pH 5.0) containing 0.075% glycine, 0.05% cupric sulfate, 0.12% acetyl thiocholine iodide (freshly prepared), and 0.0072% ethopropazine. Reactions were performed overnight at room temperature. Following incubation, the sections were rinsed five times with distilled water, incubated in 1.25% sodium sulfide solution (pH 6, freshly prepared) for 30 min at room temperature, then washed five times with distilled water, and incubated in 1% silver nitrate solution for 10 min, and finally washed with distilled water. Finally, slides were dehydrated in a graded series of ethanols and xylene, air dried and coverslipped. Images for figures were collected on an upright Olympus BX51 microscope (Olympus America).

### Fluorescence in situ hybridization (FISH)

FISH with co-immunohistochemistry was performed as previously described [6]. mRNA probe templates for human tau were generated by RT-PCR from human brain tissue and correspond to the 3’ untranslated regions of mouse Mapt (NM_001038609.1; nucleotides 1606–2588) and human Mapt (NM_016835; nucleotides 2773–3602).

### Amyloid plaque quantification

Amyloid deposition was assessed using Ab immunofluorescence labeling of 10 μm brain sections from APP/PS1 and rTgTauEC x APP/PS1 mice (as described above). Regions of interest were outlined manually on captured images using Fiji. These regions were then subjected to a macro which automatically thresholded and quantified the size and number of the plaques. Four brain sections were averaged for the mean average of each animal.

### Aβ elisa

To determine concentrations of Aβ_1–40_, 12-month-old Tg4510 mouse brains (cortex and hippocampus) were homogenized in TBS containing protease inhibitors (Complete, Roche). Concentrations of Aβ were determined using the Wako Human/Rat (Mouse) b-Amyloid (40) ELISA Kit according to the manufacturer’s protocol.

### Real-time PCR analysis

Total RNA from five rTgTauEC x APP/PS1 and three APP/PS1 mice was prepared using Trizol reagent (Sigma Aldrich). RNA integrity was assessed using the Agilent 2100 Bioanalyzer system and Agilent RNA 6000 Pico Kit (Agilent Technologies). For RT-PCR analysis, All RNA samples were transcribed into cDNA using SuperScript® III Reverse Transcriptase (Life Sciences) in a 20 μl volume. The abundance of transcripts in cDNA samples was measured by RT-PCR on Bio-Rad I-cycler with RT^2^ qPCR Primer Assay for Human APP (SABiosciences) following the manufacturer’s recommendations. The levels of APP expression were normalized to GAPDH. qPCR data were tested for statistical significance (p ≤ 0.05) using the two-tailed *t*-test.

### Western blot analysis

Brain homogenates from indicated regions were analyzed by immunoblot as previously described [6], with antibodies directed against total tau (1:5000, DAKO); human tau (Tau13, 1:2000, Covance); synapsin I (1:1000, Millipore); and β-actin (1:5000, Abcam).

### SNP analysis of mouse lines

rTgTauEC x APP/PS1 mice (N = 11) were genotyped by GeneSeek (Lincoln, NE) with the MegaMouseUniversalGenomeArray (MegaMUGA, http://csbio.unc.edu/CCstatus/index.py), which includes up to 77,800 single nucleotide polymorphism (SNP) markers.

### Statistical analysis

Data were analyzed with appropriate statistics including Student’s *t* test and one-way ANOVA followed by Tukey or Dunnett’s post-hoc test where necessary. *P* values <0.05 were considered to be statistically significant. Data are reported as mean ± SEM.

## Results

### Tau propagation is enhanced in rTgTauEC x APP/PS1 mice compared to rTgTauEC

**Figure 1:**
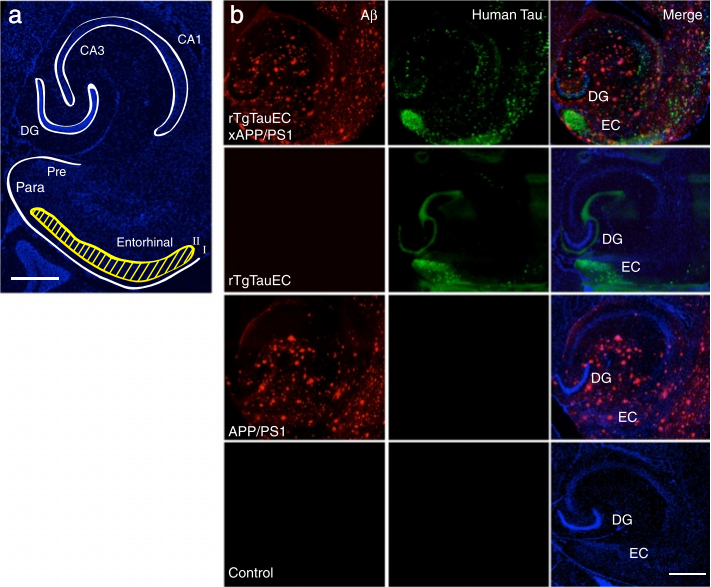
**Patterns of human tau protein and deposition of Aβ in transgenic mouse models of AD. (a)** Low-magnification view of a 16-month-old rTgTauEC horizontal brain section labeled with DAPI. Regions of interest are outlined in white; layer II of the EC, the primary expression site for the P301L transgene, is highlighted in yellow (scale bar = 500 μm). **(b)** rTgTauEC x APP/PS1 mice express human tau protein, labeled with a human-specific tau antibody Tau13 (green), and have robust deposition of amyloid plaques (anti-Aβ antibody AW7, red) at 16 months of age. DAPI staining for cell bodies appears in blue. No human tau positive neurons are observed in control (neuropsin promotor only) or APP/PS1 mice, and there is no detectable deposition of amyloid plaques in control or rTgTauEC mice. Scale bar = 500 μm.

In rTgTauEC mice, targeted overexpression of human P301L tau in the EC causes the progressive accumulation of pathological tau aggregates first in the neurons expressing the transgene, and then in synaptically connected neurons including the granule cells of DG and CA1/CA3 cells of the hippocampus [6,21]. To determine whether Aβ pathology alters tau pathology progression and enhances synaptic tau transmission in these mice, we produced a rTgTauEC x APP/PS1 mouse line [23] as described in Materials and Methods. Brain regions of interest are highlighted in white in a representative horizontal section; layer II of the EC is highlighted in yellow (Figure 1a). At 16 months, as expected, these mice show amyloid plaque deposition throughout the brain, and clear human tau immunostaining in the EC (Figure 1b). Brains of rTgTauEC x APP/PS1 animals were immunolabled and analyzed to examine the detailed pattern of human tau distribution.

**Figure 2:**
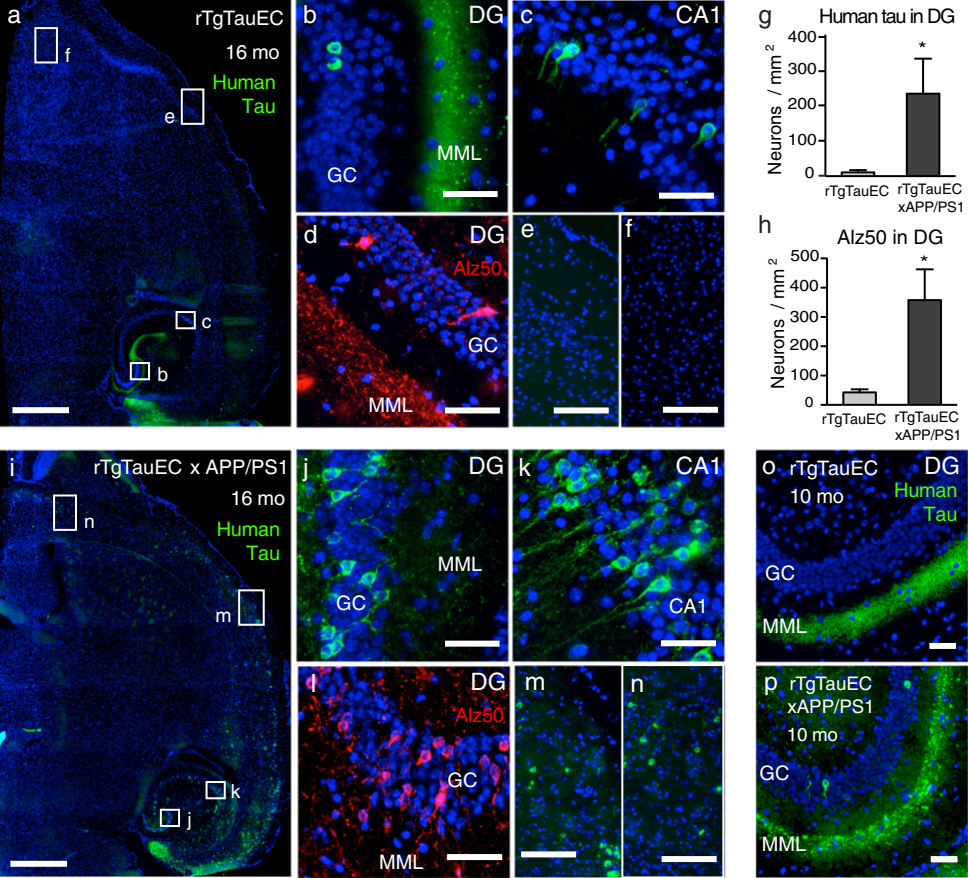
**Propagation of tauopathy along neural circuits is exacerbated by amyloid pathology.** Low magnification view of horizontal sections of 16 month old brains from **(a)** rTgTauEC and **(i)** rTgTauEC x APP/PS1 mice, labeled with a human-specific tau antibody Tau13 (green) and cell bodies stained with DAPI (blue; scale bar = 1 mm), reveal increased propagation of tauopathy in rTgTauEC x APP/PS1 mice compared with rTgTauEC mice. **(b,** **j)** Higher magnification of layers of the DG showing the middle molecular layer (MML) and granule cell layer (GC; scale bar = 50 μm); and **(c,** **k)** CA1 fields of the hippocampus (scale bar = 50 μm) show human-tau positive cells in brain regions synaptically connected to the EC. Note apparent degeneration of the MML of the DG in rTgTauEC x APP/PS1 mice. In rTgTauEC x APP/PS1 mice, but not rTgTauEC mice, human tau is observed in brain regions distal to the EC such as somatosensory cortex **(e,** **m)** and accessory olfactory areas (**f** *,* **n**; scale bars 100 μm). **(d,** **l)** Immunolabeling for Alz50 (red; scale bar 50 μm), a marker of abnormally folded tau, was evident in neurons in the GC layer of the DG and increased in rTgTauEC x APP/PS1 mice compared with rTgTauEC mice. Again, loss of Alz50-positive terminals in the MML was observed. **(g,** **h)** Quantification of human tau-positive neurons in the DG granule cells immunolabeled with Tau13 (rTgTauEC, N = 7; rTgTauEC x APP/PS1, N = 10) or Alz50 (rTgTauEC, N = 7; rTgTauEC x APP/PS1, N = 7) in 16-month-old animals. Significantly more neurons containing human tau were observed in rTgTauEC x APP/PS1 mice compared with rTgTauEC mice, indicating increased propagation in this group. **(o,** **p)** In 10-month-old mice, human tau propagation (Tau13) from the EC to the DG was observed only in rTgTauEC x APP/PS1 mice (rTgTauEC, N = 7; rTgTauEC x APP/PS1, N = 7). Values represent mean ± s.e.m; *P < 0.05.

In 16-month-old rTgTauEC mice, we found sparse but occasional human tau immunopositive inclusions in DG granule cells (Figure 2a,b), confirming the previously observed infrequent propagation of human tau in rTgTauEC mice younger than 24 months [6,21]. By contrast, rTgTauEC x APP/PS1 mice had substantially more human tau positive DG granule cells (approximately 20-fold increase relative to rTgTauEC, *p* < 0.05) (Figure 2i,g,j). Human tau that accumulated in granule cells of the DG was also positive for Alz50, an antibody that recognizes abnormally folded tau (Figure 2d,h,l). Furthermore, more human tau was also detected in CA1 hippocampal neurons in rTgTauEC x APP/PS1 than in rTgTauEC mice (Figure 2c,k), suggesting that the presence of APP/PS1 accelerates the interneuronal transfer of tau from the entorhinal cortex into neurons in DG and CA1. Previously we observed no overt differences in propagation between groups [23], but this is likely due to the small number of animals examined in that study, and they were not studied quantitatively. Here, the larger numbers of animals per group allowed for quantification of clear differences between the mouse lines. No difference in human tau protein expression was detected between the rTgTauEC and rTgTauEC x APP/PS1 genotypes (Additional file 1: Figure S1). As expected, no human tau immunoreactivity was observed in control or APP/PS1 mice (Figure 1).

Next we examined whether human tau propagates even further to more distal brain regions in rTgTauEC x APP/PS1 mice. Strikingly, at 16 months, human tau-positive neurons were also found in the somatosensory cortical area (Figure 2m) and accessory olfactory areas (Figure 2n) where no human tau was observed in rTgTauEC mice (Figure 2e,f). While most of the crossed mice showed marked enhancement of propagation to the hippocampus, about half of the mice (6 of 13 examined) showed this effect to a much greater extent. Propagation of tau to these distant brain regions may be facilitated by direct tau input from neurons in the EC or, more likely, indirectly across multiple synapses.

To further explore the hypothesis that the presence of amyloid pathology induces acceleration of trans-synaptic tau propagation, we next examined brains of younger rTgTauEC x APP/PS1 animals. In 10-month-old mice, human tau pathology, including dystrophic neurites, was detected in the DG in rTgTauEC x APP/PS1 mice (3 of 7 examined), but not in any rTgTauEC (Figure 2o,p). These observations suggest that the presence of amyloid pathology enhances the trans-synaptic transfer of tau *in vivo*.

The variability observed in tau propagation in the rTgTauEC x APP/PS1 mice raised the possibility of a genetic influence on prion-like spread of tau pathology. Indeed, although all rTgTauEC x APP/PS1 mice were highly similar genetically and shared the (FVBxB6)F1 background, the B6.Tg(EC-tTA) transactivator line was not completely congenic and carried DBA/2 J passenger genes. More than 20,000 markers distinguished B6 mice from DBA/2 J and C3H/HeJ, which potentially contaminated the Tg(EC-tTA) and Tg(APP/PS1) B6 congenic strains. However, SNP genotyping provided no evidence of a non-B6 derived region in the rTgTauEC x APP/PS1 mice that co-segregated with the tau propagation phenotype, indicating that the variability in tau propagation among mice is unlikely to be due to a Mendelian genetic trait.

### The presence of amyloid accelerates synaptic alterations in the EC of rTgTauEC mice

Previous characterization of the rTgTauEC mice noted a marked degeneration of tau-positive terminals in the middle molecular layer (MML) of the DG after 24 months, suggesting degeneration of perforant pathway axons that arise from EC neurons [6]. In the present study, the MML appeared fairly unremarkable at 16 months in the rTgTauEC mice, whereas rTgTauEC x APP/PS1 mice showed robust degeneration of the tau-positive terminals in this region (Figure 2b,j), suggesting a profound loss of EC inputs.

**Figure 3:**
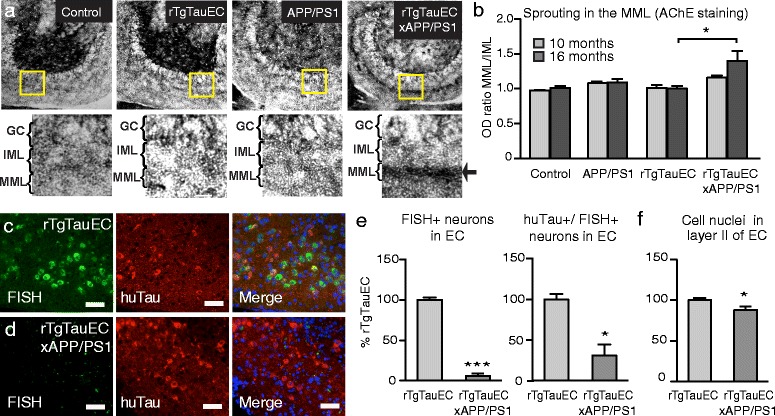
**Tauopathy-induced neurodegeneration is enhanced by amyloid deposition.** **(a)** Histochemistry of AChE fibers in the DG shows deafferentation-induced reorganization in the MML in 16-month-old rTgTauEC x APP/PS1 mice. Increased AChE staining in the MML is indicated by the arrow. **(b)** Comparing the ratio of optical densities between MML and IML demonstrates that the sprouting of AChE fibers is significant at 16 months of age in rTgTauEC x APP/PS1 mice compared to rTgTauEC, but no synaptic reorganization is evident in 10-month-old animals (10 and 16 month old animals, N = 3). **(c,** **d)** High magnification views of the EC with FISH showing human tau mRNA (green) and immunostained for human tau protein (Tau13, red; scale bar = 50 μm). Transgene expression was robust in layer II of the EC in rTgTauEC mice, but nearly absent in rTgTauEC x APP/PS1 mice, despite strong labeling for human tau protein. **(e)** (Left) Quantification of FISH labeling reveals that rTgTauEC x APP/PS1 mice have a significant reduction in transgene expressing cells in the EC compared with rTgTauEC mice (rTgTauEC, N = 3; rTgTauEC x APP/PS1, N = 6). (Right) The EC of rTgTauEC x APP/PS1 mice also contained fewer cells that were positive for both human tau mRNA and tau protein. **(f)** Layer II of the EC of rTgTauEC x APP/PS1 mice contained significantly fewer cells than rTgTauEC mice. Values represent mean ± s.e.m; *P < 0.05, ***P < 0.001.

In classical deafferentation paradigms, loss of perforant pathway axons from the EC leads to deafferentation of their terminal zone in the MML of the DG [14]. Subsequently, neighboring acetylcholinesterase (AChE) containing fibers, located in the inner molecular layer react to the deafferentation by sprouting aberrantly into the MML [17]. The amount of AChE fiber sprouting into the MML correlates with the severity of perforant pathway degeneration and has been detected in 21-month old and more robustly in 24-month old rTgTauEC mice [20]. Visualization of these fibers was acheived by histochemical staining for AChE, as previously described [7,20]. In the present study, we compared sprouting of AChE-positive fibers into the MML in 10- and 16-month-old rTgTauEC x APP/PS1, rTgTauEC, APP/PS1, and non-transgenic control mice. In 10-month-old mice, no overt sprouting in any of the mouse lines was observed. However, 16-month old rTgTauEC x APP/PS1 mice showed significantly stronger AChE staining in the MML than all the other mouse lines (Figure 3a,b). At this age no overt loss of the pre-synaptic protein synapsin was observed in either the EC or the hippocampus of the rTgTauEC x APP/PS1 mice (Additional file 2: Figure S2). We also found increased expression of GFAP in the EC of rTgTauEC x APP/PS1 and APP/PS1 mice compared to rTgTauEC and controls, particularly in the vicinity of ThioS-positive amyloid plaques (Additional file 3: Figure S3), indicating widespread astrogliosis in agreement with previous studies of the APP/PS1 line [18]. Increased neuroinflammation in rTgTauEC x APP/PS1 mice may therefore contribute to enhanced tau propagation and EC degeneration relative to rTgTauEC mice. These data indicate that synergy between APP/PS1 and human P301L tau enhances degeneration of perforant path inputs to the DG.

### Co-occurrence of amyloid deposition in rTauEC line leads to a profound loss of tau expressing entorhinal cortex neurons

We determined the expression of the human tau transgene in the rTgTauEC and rTgTauEC x APP/PS1 lines using fluorescent *in situ* hybridization (FISH) of 10 μm horizontal brain sections from 10- and 16-month-old animals. At 10 months of age, as expected tau transgene expression displayed a restricted distribution to the medial EC, with limited expression in the neighboring pre- and para-subiculum in both rTgTauEC and rTgTauEC x APP/PS1 mice (Additional file 4: Figure S4). At 16 months of age, human tau transgene expression in the medial EC was robust in rTgTauEC mice (Figure 3c). However, unexpectedly, rTgTauEC x APP/PS1 mice displayed a nearly complete reduction in the number of FISH-positive cells compared with mice expressing rTgTauEC only (Figure 3d, e). No FISH-positive cells were found in the DG of either mouse line (Additional file 5: Figure S5), as expected. Co-labeling of the same brain sections with an anti-human tau antibody (Tau13) revealed strong immunoreactivity in the EC in both mouse lines. Closer examination of EC neurons revealed that a proportion of neurons were immunoreactive for Tau13 but negative for human tau mRNA (Figure 3c), confirming previous findings that tau protein is able to transfer between neurons in this mouse model [6]. However, significantly fewer EC neurons were positive for both human tau mRNA and protein in the rTgTauEC x APP/PS1 compared to rTgTauEC mice (Figure 3e), and there was overall a loss of >90% of tau mRNA expressing neurons. Quantification of the total number of cells in layer II of EC revealed significantly fewer cells in rTgTauEC x APP/PS1 mice compared with rTgTauEC mice (Figure 3f), suggesting that enhanced cell death occurs in mice with both tau and Aβ pathologies. The magnitude of neuronal loss appeared smaller when all EC layer II cells, and not only the tau-containing cells, are quantified since human tau mRNA expression is limited to a subset of neurons (approximately 10 – 15%) in layer II of the EC [6]. Together these data demonstrate a profound degeneration of transgene-expressing neurons in the rTgTauEC x APP/PS1 mice.

### Tau expression in the entorhinal cortex impacts characteristics of amyloid deposition

**Figure 4:**
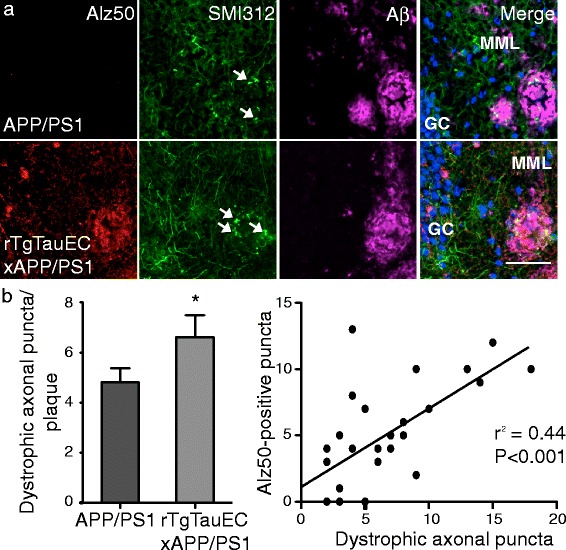
**The presence of human tau exacerbates amyloid plaque-induced axonal dystrophy.** **(a)** Amyloid plaques in the MML were visualized with the antibody AW7, and were co-immunostained with SMI312, an axonal marker, and with the tau-specific Alz50 antibody (scale bar = 50 μm). Dystrophic axonal puncta are indicated by white arrows. **(b)** Quantification of SMI312-positive puncta within amyloid plaques revealed significantly more dystrophic axons in rTgTauEC x APP/PS1 mice compared with APP/PS1 mice. Further analysis of individual amyloid plaques showed a significant correlation between the numbers Alz50-positive and SMI312 axonal puncta (APP/PS1, N = 4; rTgTauEC x APP/PS1, N = 5). Values represent mean ± s.e.m; *P < 0.05.

Axonal dystrophy in the vicinity of amyloid plaques, as a result of amyloid-beta toxicity, has been described in both mouse models [4] and human brain affected by AD [19]. To examine whether the presence of human tau enhances amyloid toxicity, we quantified dystrophic axons within amyloid plaques in the MML in 16-month-old APP/PS1 and rTgTauEC x APP/PS1 mice. Axonal dystrophies were immunolabeled using an antibody against phosphorylated neurofilament proteins (SMI312) that are present in dystrophic axons, and amyloid plaques were stained with the anti-amyloid antibody AW7 (Figure 4a). The presence of abnormally folded tau was detected with Alz50. Counting SMI312-positive punctae within amyloid plaques we detected a significant increase in axonal dystrophies in the MML of rTgTauEC x APP/PS1 compared with to APP/PS1 mice (Figure 4b), and in rTgTauEC x APP/PS1 mice the amount of abnormally folded tau correlated linearly (r^2^ = 0.44) with the number of dystrophic punctae in a plaque (Figure 4b). Taken together, our data suggest that the presence of pathological tau exacerbates amyloid-induced axonal defects.

### Presence of tau may impact amyloid plaque burden

**Figure 5:**
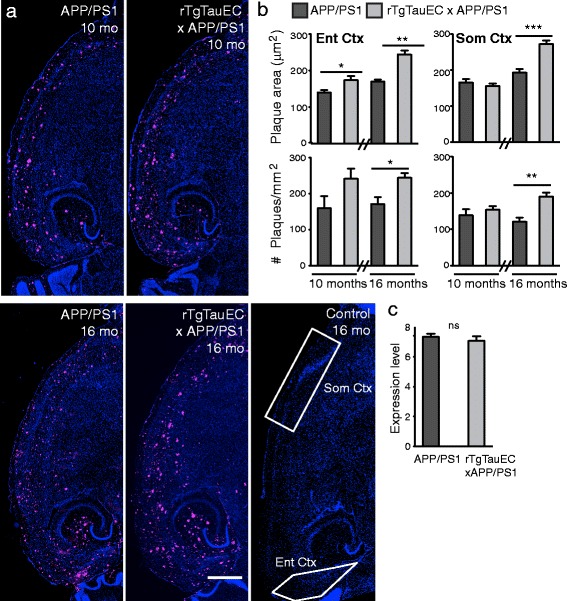
**Expression of human tau increases amyloid plaque burden.** **(a)** Low-magnification view of horizontal brain sections from APP/PS1 and rTgTauEC x APP/PS1 mice at 10 months of age (Top) and APP/PS1, rTgTauEC x APP/PS1 and control mice at 16 months of age (Bottom), immunostained with AW7 antibody to visualize amyloid plaques. Regions of interest are outlined in white on control (neuropsin promotor only) image (scale bar = 1 mm). **(b)** Top: Quantification of amyloid plaque area revealed significantly larger plaques in rTgTauEC x APP/PS1 mice compared with APP/PS1 mice the EC in 10- and 16 months of age, and in the somatosensory cortex at 16 months of age. Bottom: The number of amyloid plaques was also increased in 16-month-old rTgTauEC x APP/PS1 mice in both the EC and somatosensory cortices (10 months: APP/PS1, N = 7; rTgTauEC x APP/PS1, N = 7. 16 months: APP/PS1, N = 4; rTgTauEC x APP/PS1, N = 11). **(c)** Expression of human APP transgene was assessed by qPCR. No differences in APP mRNA were observed in 16-month-old APP/PS1 vs. rTgTauEC x APP/PS1 mice (APP/PS1, N = 3; rTgTauEC x APP/PS1, N = 5). Values represent mean ± s.e.m; *P < 0.05, **P < 0.01, ***P < 0.001.

Since human P301L tau appeared to aggravate amyloid toxicity, we next asked whether pathological tau also enhances amyloid plaque formation. In horizontal brain sections of 10- and 16-month-old APP/PS1 and rTgTauEC x APP/PS1 mice, we immunolabeled plaques using the anti- Aβ antibody AW7 and determined amyloid plaque number and size in both the EC and the somatosensory cortex (Figure 5a). In 10-month-old mice, when compared to APP/PS1 mice, rTgTauEC x APP/PS1 mice showed an increase in plaque area (significant, p = 0.022) but plaque number did not reach significance (p = 0.087) in the EC, and no change was observed in somatosensory cortex (Figure 5b). At 16 months of age, both entorhinal and somatosensory cortex, rTgTauEC x APP/PS1 mice had significantly more amyloid plaques than APP/PS1 mice. Image analysis suggested that these plaques were also significantly larger (Figure 5b). By performing qPCR on brain tissue from adjacent brain sections we confirmed that the difference in amyloid burden between rTgTauEC x APP/PS1 and APP/PS1 mice did not originate from differential expression of the human APP transgene (Figure 5c).

## Discussion

In AD, both NFT and plaques appear in the cortex, but whether this is co-occurrence of two separate lesions or if there is a pathophysiological interaction between plaques and tangles is unknown. On the basis of well-established patterns of human neuropathological stages of AD, we considered two possibilities: that the presence of plaques in the cortex might accelerate or enhance the “spread” of tangles from the medial temporal lobe to the cortex, or that they are independent events co-occurring during disease progression. The present findings show that the presence of amyloid pathology exacerbates the spread of tau from neurons in the EC to synaptically-connected brain regions. The mouse models presented in this study permitted us to visualize the progression of tau pathology in the presence or absence of widespread Aβ plaques, therefore closely recapitulating conditions occurring early in AD. We examined the consequence of the co-occurrence of these lesions on tau propagation and toxicity. Our results indicate that the presence of Aβ deposition 1) significantly promotes trans-neuronal propagation of tau pathology, and 2) accelerates degeneration of human tau-expressing neurons. Furthermore, accumulation of human tau also impacts amyloid pathology, by exacerbating plaque-related axonal dystrophy and increasing Aβ plaque burden. Together, our data imply a synergistic reinforcement between tau and amyloid pathology in AD, with a dramatic order of magnitude increase in tau propagation and toxicity in the presence of Aβ.

In rTgTauEC x APP/PS1 mice, tau-positive neurons in the DG were increased approximately 20-fold compared to modest levels of tau propagation in the parental rTauEC line, at 16 months of age. Furthermore, 16 month old rTgTauEC x APP/PS1 mice demonstrate propagation of tau aggregates to more distal regions, such as the accessory olfactory areas, olfactory bulb and frontal cortices, areas only marginally involved even at 24 months in rTauEC mice. Thus, it appears that the presence of amyloid accelerates the interneuronal transfer of tau. The mechanism of this enhancement of tau related phenotypes is not clear. For example, the presence of amyloid may alter the regulated release of tau into extracellular space [22,29] or promote its specific uptake [28] by synaptically-connected neurons, and these actions may be further exacerbated by neuroinflammation. We conclude that the presence of neocortical amyloid deposits is part of a causal chain that leads to augmentation of the NFT trans-synaptic propagation phenotype.

Equally importantly, profound loss of EC layer II neurons was observed in 16 month old rTgTauEC x APP/PS1 mice, again in contrast to no detectable loss of these tau expressing neurons in the parenteral rTauEC line at this age. Taken together, our current data suggest that pathological tau and amyloid synergistically enhances EC cytotoxicity and subsequent perforant path degeneration in AD.

In addition to asking whether amyloid pathology affects the progression of tau pathology, we also compared the rTgTauEC x APP/PS1 and APP/PS1 mouse lines to assess the impact of human tau on amyloid deposition. Although the amyloid cascade hypothesis postulates that Aβ is the initiator of the pathological cascade of AD [9], very recent meta-analyses of pathophysiological biomarkers of AD suggest that Aβ and tau may be partly independent of each other and interact synergistically [13,5]. Indeed, in the present study we found that the presence of human tau exacerbated plaque-induced neuritic dystrophy, indicating that tau can enhance amyloid deposition and toxicity. In preliminary studies, we also measured Aβ concentrations in brains of rTg4510 mice, a related mouse line that expresses human P301L tau throughout the forebrain and displays robust NFT pathology [26]. We found that the concentration of endogenous mouse Aβ was significantly higher in rTg4510 mice, in both the cortex and the hippocampus (Additional file 6: Figure S6) compared to wild-type mice. These data suggest that the presence of pathogenic tau may enhance accumulation of Aβ peptide, in concert with earlier work that found increased amyloid burden in mice overexpressing both human mutant APP and tau [25]. Although the mechanisms by which tau might influence Aβ deposition require further investigation, our data demonstrating increased Aβ concentrations in a tauopathy mouse line suggest that the presence of pathological tau can increase Aβ.

## Conclusions

Several previous observations have suggested a link between tau and Aβ in mouse models [24,25,2,8] - for example, injection of tau overexpressing mice with brain extract from APP23 mice increased tau deposition; and injection of Aβ_1–42_ fibrils into P301L tau-expressing mice induced NFT deposits. the current results extend these data, and suggest a specific effect of Aβ on tau propagation and toxicity, in a way that directly models the disease progression in human patients. Neuropathological studies suggest that although entorhinal NFT are nearly universal after the age of 65 – and detectable as early as age 40 [3] – with increased NFT number and wider distribution with increasing age and the co-occurrence of Aβ deposits in the cortex. Together, these data support a model in which the presence of amyloid in neocortical projection target zones accelerates the propagation of tau across neural circuits, as well as enhancing the toxicity of tau as assessed by neuritic changes and even neuronal death, in agreement with hypotheses derived from observation of post-mortem human brain. These results thus contrast with predictions of the linear amyloid hypothesis in which amyloid toxicity causes tau related changes; instead, these data favor a model in which amyloid acts synergistically to accelerate tau propagation and toxicity, which, in this model, is an independently-driven process. The rTgTauEC x APP/PS1 mice thus provide a model in which to further test tau-Aβ interactions, and to better understand underlying mechanisms whereby tau propagation is accelerated.

## Additional files

Additional file 1: Figure S1. Expression of human tau in rTgTauEC rTgTauEC x APP/PS1 mouse lines. Additional file 2: Figure S2. Expression of the synaptic protein synapsin I in the various mouse lines. Additional file 3: Figure S3. Immunohistochemical analysis of astrocyte activation in the mouse lines. Additional file 4: Figure S4. FISH for human tau transgene expression in 10 month transgenic mice. Additional file 5: Figure S5. FISH for human tau transgene expression outside the EC in 16 month old mice. Additional file 6: Figure S6. Aβ quantification in brains of mice overexpressing mutant human tau.
